# Elevated expression of caspase-3 inhibitors, survivin and xIAP correlates with low levels of apoptosis in active rheumatoid synovium

**DOI:** 10.1186/ar2603

**Published:** 2009-01-27

**Authors:** Anak ASSK Dharmapatni, Malcolm D Smith, David M Findlay, Christopher A Holding, Andreas Evdokiou, Michael J Ahern, Helen Weedon, Paul Chen, Gavin Screaton, Xiao N Xu, David R Haynes

**Affiliations:** 1 Discipline of Pathology, School of Medical Sciences, Faculty of Health Sciences, University of Adelaide, North Terrace, Adelaide, 5005 South Australia, Australia; 2 Rheumatology Research Unit, Repatriation General Hospital, Daws Road, Adelaide, 5041 South Australia, Australia; 3 Discipline of Orthopaedics and Trauma, School of Medicine, Faculty of Health Sciences, University of Adelaide and Hanson Institute, Frome Road, Adelaide, 5005 South Australia, Australia; 4 MRC Human Immunology Unit, Weatherall Institute of Molecular Medicine, John Radcliffe Hospital, Oxford OX3, UK; 5 Hammersmith Hospital, Du Cane Road, London W12 0NN, UK

## Abstract

**Introduction:**

Tumour necrosis factor-related apoptosis-inducing ligand (TRAIL) is a tumour necrosis factor (TNF) family member capable of inducing apoptosis in many cell types.

**Methods:**

Using immunohistochemistry, terminal deoxynucleotidyl transferase biotin-dUTP nick end labelling (TUNEL) and real-time PCR we investigated the expression of TRAIL, TRAIL receptors and several key molecules of the intracellular apoptotic pathway in human synovial tissues from various types of arthritis and normal controls. Synovial tissues from patients with active rheumatoid arthritis (RA), inactive RA, osteoarthritis (OA) or spondyloarthritis (SpA) and normal individuals were studied.

**Results:**

Significantly higher levels of TRAIL, TRAIL R1, TRAIL R2 and TRAIL R4 were observed in synovial tissues from patients with active RA compared with normal controls (p < 0.05). TRAIL, TRAIL R1 and TRAIL R4 were expressed by many of the cells expressing CD68 (macrophages). Lower levels of TUNEL but higher levels of cleaved caspase-3 staining were detected in tissue from active RA compared with inactive RA patients (p < 0.05). Higher levels of survivin and x-linked inhibitor of apoptosis protein (xIAP) were expressed in active RA synovial tissues compared with inactive RA observed at both the protein and mRNA levels.

**Conclusions:**

This study indicates that the induction of apoptosis in active RA synovial tissues is inhibited despite stimulation of the intracellular pathway(s) that lead to apoptosis. This inhibition of apoptosis was observed downstream of caspase-3 and may involve the caspase-3 inhibitors, survivin and xIAP.

## Introduction

Decreased apoptosis has been proposed as a possible factor that contributes to the hyperplasia of the synovial membrane and accumulation of inflammatory cells observed in the synovitis of patients with active rheumatoid arthritis (RA) [1,2]. Inducing apoptosis in these synovial cells has the potential to reduce the disease severity and progression similar to that suggested previously for apoptosis via the FAS-FAS ligand pathway [3,4].

Tumour necrosis factor-related apoptosis-inducing ligand (TRAIL) is a member of the tumour necrosis factor (TNF) family and a type II membrane bound cytokine that is expressed by many cell types [5,6]. Although TRAIL mainly mediates apoptosis, like many other TNF family members, it has many other roles including regulation of endothelial nitric oxide synthase and the innate immune system [7,8]. In relation to apoptosis TRAIL has two types of receptors that differ in their ability to either initiate or inhibit TRAIL-mediated apoptosis [9]. TRAIL R1 (death receptor 4) and TRAIL R2 (death receptor 5) induce apoptotic cell death. The second type of TRAIL receptors act as decoy receptors and these are TRAIL R3 (DcR1, decoy receptor 1), TRAIL R4 (DcR2, decoy receptor 2) and osteoprotegerin (OPG) [10].

TRAIL and TRAIL death receptors form a complex, which transmits an apoptotic signal via the Fas associated death domain (FADD). This leads to activation of caspase-8 or other initiator caspases, which in turn activate downstream caspases (such as caspase-3, 9, 6 and 7) that cause cell death.

Inhibition of apoptosis mediated by TRAIL could occur upstream or downstream of the pathway. At the upstream levels the inhibition could result from the expression of TRAIL decoy receptors, while at the intracellular signalling level proteins capable of inhibiting caspase activation, such as FLIP (flice inhibitory protein) [11], that blocks initiator caspase (caspase-8) and IAP (inhibitor of apoptosis protein) family members [12], that block effector caspase (caspase-3) further downstream, could potentially inhibit apoptosis.

Several studies have reported on the importance of TRAIL and TRAIL receptor expression in inducing or inhibiting apoptosis [13-16]. Some studies have shown that TRAIL and its receptor, TRAIL R2, are expressed in the synovial tissues of RA patients [17,18] and TRAIL R2 is highly expressed in synovial cells in culture [18-21]. TRAIL gene therapy has been reported to inhibit development of arthritis in a collagen-induced mouse model [17,22]. In addition, an agonistic monoclonal antibody that binds to the TRAIL death receptor, TRAIL R2, has been reported to induce apoptosis in RA synovial fibroblasts [18,19]. However, none of the studies comprehensively investigated TRAIL and all its receptors in the synovial tissue from patients with various types of arthritis.

In addition to TRAIL and its receptor interaction, recent evidence suggests that intracellular regulators such as FLIP, caspases [23], members of the Bcl2 family [24] and tumour suppressor proteins such as p53 are often central in determining whether apoptosis occurs in particular cells [11]. Recently, survivin, a member of the IAP family, has been reported to be elevated in serum in RA, with high levels correlating with joint erosion in active RA [25].

Many of the previous studies [18,19,21,26,27] have focused on investigating the expression of TRAIL and TRAIL receptors using synovial fibroblasts in culture. However, this population of cells may not be representative of the inflammatory cells, such as lymphocytes and cells of the macrophage/monocyte lineage. Therefore, the present study investigated the expression of TRAIL and each of the TRAIL receptors in synovial tissues *in situ *from a range of arthritic conditions, including RA and OA. The expression of intracellular pro- and anti-apoptotic molecules was simultaneously investigated with the extent of apoptosis in pathogenic and normal human synovial tissues.

## Materials and methods

### Patients and tissue preparation

Synovial tissue samples were obtained from patients at the time of knee arthroscopy or total knee replacement (OA patients) at the Rheumatology unit at the Repatriation General Hospital, Adelaide, South Australia. Normal synovial tissues were obtained from patients attending sports medicine clinics with unexplained knee pain. All patients with active RA or spondyloarthropathy (SpA) had active joint inflammation. Patients with inactive RA were in remission after successful disease modifying antirheumatic drug (DMARD) treatment for active RA and were undergoing a further knee joint arthroscopy for routine follow-up.

Informed consent was obtained from the patients before the procedures and the study was approved by the Repatriation General Hospital Human Ethics Committee. All patients diagnosed with RA fulfilled the 1987 revised criteria of the American College of Rheumatology (ACR) [28]. Osteoarthritis (OA) patients fulfilled the criteria by Altman and colleages [29] and SpA patients fulfilled the European Spondyloarthropathy Study Group criteria [30]. Characteristics of patients are summarised in Table 1.

**Table 1:** Demographic of patients

Patient's	Active RA	Inactive RA	OA	SpA	Normal
Number	21	9	7	12	17
Age (years) (mean;SEM)	65.33 (3.32)	72.33 (2.35)	69.7 (2.34)	48.72 (5.66)	33.41 (2.45)
Disease duration (months) (mean;SEM)	3.76 (.50)	21.77 (3.63)	NA	72.9 (19.32)	NA
Gender (F/M)	11 (10)	3 (6)	1 (6)	5 (7)	7 (10)

DMARDs	17 NSAIDs	2 MTX	6 NSAIDs	8 NSAIDs	NA
	2 Hydroxy	1 Hydroxy	1 Panadeine-F	2 SSZ	
	1 Pred	4 Gold		1 SSZ/AZA/pred	
	1 SSZ	1 SSZ		1 SSZ/NSAIDs	
		1 gold/MTX			

CRP (mg/L) (mean;SEM)	67.67 (13.5)	4.7 (1.03)	NA	43.45 (7.65)	NA
RF	11 positive	5 positive	NA	NA	NA
Erosions	6 positive	2 positive	NA	7 positive	NA
DAS 28 (mean;SEM)	5.47 (0.22)	1.08 (0.24)	NA	NA	NA

AZA = azathioprine; CRP = C-reactive protein; DAS28 = disease activity score 28; DMARD = disease modifying antirheumatic drug; F = female; Gold = intramuscular sodium aurothiomalate injections; Hydroxy = hydroxychloroquine; M = male; MTX = methotrexate; NA = not available; NSAIDs = nonsteroidal anti-inflammatory drugs; OA = osteoarthritis; Panadeine F = paracetamol/codeine combination; Pred = prednisolone; RA = rheumatoid arthritis; SEM = standard error of the mean; SpA = spondyloarthropathies; SSZ = sulphasalazine.

Clinical and laboratory data were collected from all RA patients including tender and swollen joint count, patient assessment of pain and global score of disease activity and physician score of global disease activity (all on visual analogue scales). In addition, C-reactive protein (CRP), erythrocyte sedimentation rate (ESR), rheumatoid factor levels (RF), disease activity score (DAS28), as well as x-rays of the hands and feet were assessed for the presence of joint erosions. Frozen sections and formalin-fixed paraffin-embedded tissue were prepared from the synovial biopsies. When available, an additional synovial biopsy was placed into RNAlater (Ambion, Foster City, CA, USA) and stored at 4°C until RNA extraction was performed.

### Monoclonal antibodies

Recombinant human TRAIL (375-TL/CF) and monoclonal antibodies directed against TRAIL (MAB687, immunoglobulin (Ig) G1), TRAIL receptor 3/DcR1 (M430, IgG1), rabbit anti-human survivin (AF886, polyclonal IgG) and anti-human x-linked inhibitor of apoptosis protein (xIAP) (MAB8221, IgG1) were purchased from R&D Systems, Inc. (Minneapolis, MN, USA). Monoclonal antibodies against TRAIL receptor 1/TRAIL R1 (M271, IgG2a), TRAIL receptor2/TRAIL R2 (M413, IgG1) and TRAIL receptor 4/TRAIL R4 (M444, IgG1) were gifts from Amgen (Thousand Oaks, CA, USA). Monoclonal antibodies against TRAIL R2 were used as described previously [31]. Monoclonal antibodies against cleaved caspase-3 (cat no # 9664) were purchased from Cell Signaling Technology Inc. (Beverley, MA, USA) and monoclonal antibodies against cleaved caspase-8 (AP1013) were purchased from Calbiochem (Darmstad, Germany). Anti-human CD3 monoclonal antibodies (Clone Sk7) were used to detect T lymphocytes, anti-CD22 antibodies (clone Mc 64-12) were used to detect B lymphocytes and anti-CD68 antibodies (clone EBMII) were used to detect macrophages and were all purchased from Dako (Botany, NSW, Australia). Anti-human CD55 antibodies (MCA 1614 GA) were used to detect synovial lining fibroblasts and were obtained from Serotec (Kidlington, Oxford, UK). Frozen sections were used for immunostaining with all antibodies except for those studies using TRAIL R2 and TRAIL R3 in which formalin-fixed paraffin-embedded material was used.

### Immunohistochemical detection

For immunoperoxidase staining a three-step immunohistochemical detection was performed as described previously [32]. Staining for TRAIL, TRAIL receptors, cleaved caspases, xIAP and survivin were performed at the same time in all synovial tissues to eliminate day-to-day variability of the staining. Negative controls consisted of omission of the primary antibodies, and the use of isotype-matched antibody controls (1B5 for IgG1, 1D4.5 for IgG2a and normal rabbit serum for polyclonal antibody) [33]. Positive controls were tissues known to be positive for TRAIL and TRAIL receptors (normal colon, adenoma and carcinoma of the colon). Specificity of TRAIL staining was confirmed by antibody absorption, using recombinant human TRAIL, according to a method published previously [34].

### Double staining immunohistochemistry

Double staining was performed according to a method published previously [34] to identify the cell lineages that express TRAIL, TRAIL R1 and TRAIL R4. Antibodies directed against CD68 were used to detect macrophage lineage cells, CD55 for synovial fibroblasts in the synovial lining layer, CD22 for B cells and CD3 for T-lymphocyte lineage cells.

### Detection of apoptosis by TUNEL staining

Terminal deoxynucleotidyl transferase biotin-dUTP nick end labelling (TUNEL) staining was performed on frozen synovial tissues using a TUNEL detection kit (Roche Diagnostics, Castle Hill, NSW, Australia). As a positive control, synovial tissue was pretreated with 1 μg/mL DNA-ase for 10 minutes at room temperature before TUNEL detection. The negative control was synovial tissue incubated with label solution only according to the manufacturer's instructions.

### Semiquantitative scoring

A semi-quantitative (SQA) scoring system was carried out by two "blinded" observers (DH and AASSK) to evaluate the percentage of cell staining as the results of immunohistochemistry and TUNEL staining, using a score of 0 to 4, as described previously [35]. A score of 0 indicated that there were 0 to 10% positive cells, 1 indicated 11 to 25% positive cells, 2 indicated 26 to 50%, 3 indicated 51 to 75% and 4 indicated more than 75% positive cells.

### RNA extraction and cDNA synthesis

Total RNA was isolated from tissue after homogenisation with 1 mL/100 mg tissue TRIzol (Invitrogen Life Technologies, Carlsbad, CA, USA), according to the manufacturer's recommendations. One microgram total RNA was reverse transcribed using 250 ng random hexamer (Geneworks, Adelaide, SA, Australia) and 200 Units Superscript III Reverse Transcriptase as per the manufacturer's recommendations.

### Real-time PCR

Real-time PCR was performed using Platinum SYBR Green qPCR Supermix-UDG (Invitrogen Life Technologies, Carlsbad, CA, USA) as per the manufacturer's recommendations. Amplification was carried out in a Rotor-Gene 3000 (Corbett Life Science, Mortlake, NSW, Australia) with SYBR green detection and melt curve analysis. Oligonucleotide primers used have been described previously, and are specific for caspase-3 [36], survivin [37] and xIAP [38]. The endogenous reference gene hARP [39] was used to normalise C_t _data obtained from the genes investigated. Reaction mixtures contained 10 ng cDNA, Platinum SYBR Green qPCR Supermix-UDG, 300 nM each of forward and reverse primer and diethyl pyrocarbonate treated water to a final volume of 15 μL. All samples were investigated in triplicate and the melting curves obtained after each PCR amplification confirmed the specificity of the SYBR green assays. Relative expression of the target genes in the studied samples was obtained using the difference in the comparative threshold (ΔΔC_t_) method [40].

### Statistical analysis

Statistical analysis was performed using SPSS version 11.5 (Chicago, IL, USA). The Mann Whitney-U test was used to compare mean rank of the SQAs between two groups and Kendall's tau_b test was used for the correlation between two parameters examined with p < 0.05 accepted as indicating statistical significance.

## Results

### Expression of TRAIL and TRAIL receptors

**Figure 1:**
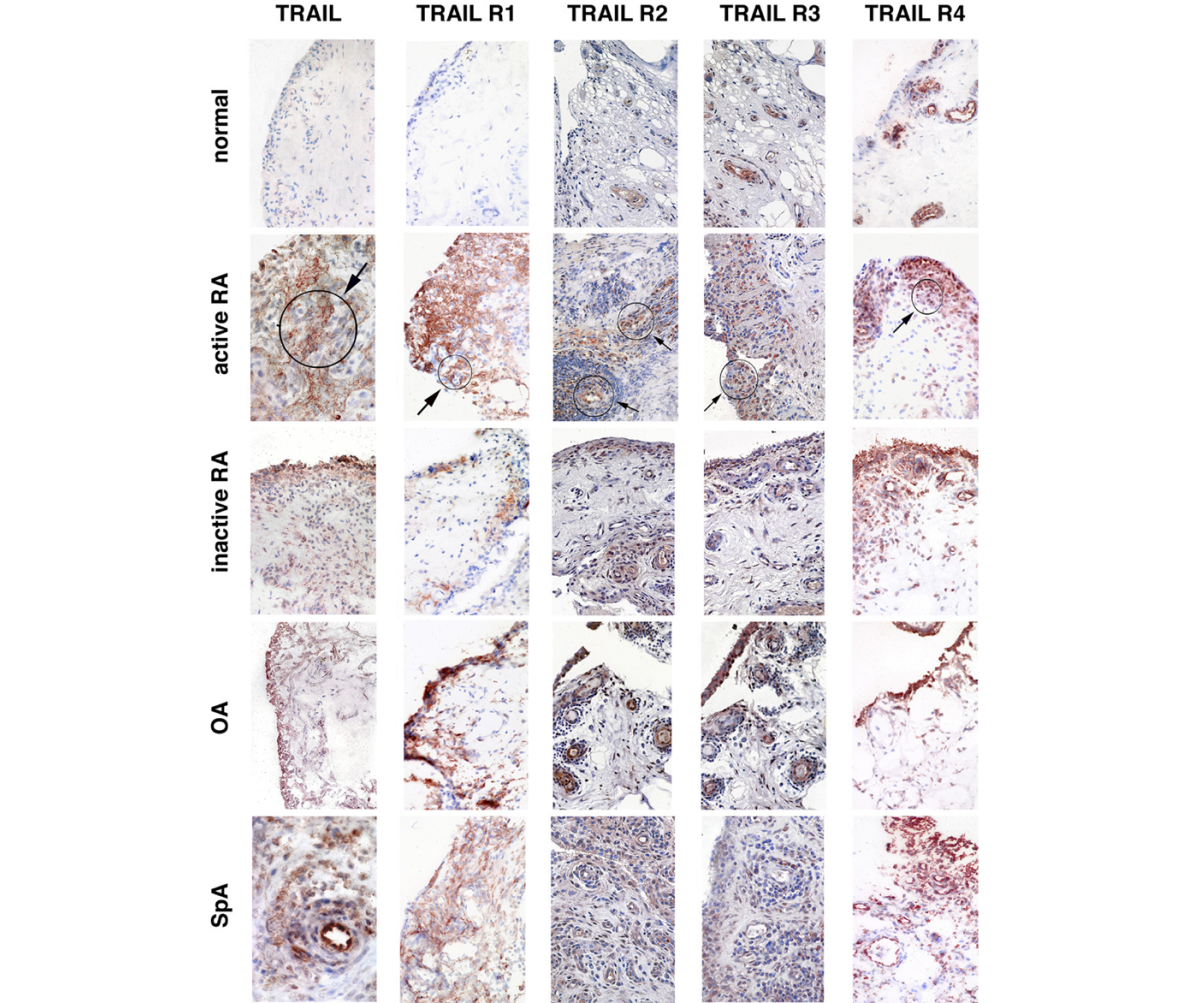
TRAIL, TRAIL R1, TRAIL R2, TRAIL R3 and TRAIL R4 expression pattern. Positive cells are shown in red staining. Staining pattern in active RA synovial tissue demonstrated in the area indicated by arrows. Magnification × 200. Synovial tissues were obtained from normal (1st row), active rheumatoid arthritis (RA) (2nd row), inactive RA (3rd row), osteoarthritis (OA) (4th row) and spondyloarthropathy (SpA) patients (5th row). TRAIL = tumour necrosis factor related apoptosis inducing ligand. Regions of interest in some panels are indicated by the circles and arrows and discussed in the text.

TRAIL expression was significantly higher in the synovial tissues from active RA and SpA (p < 0.05) compared with normal synovial tissues (Table 2; Figure 1). In addition, a marked increase in the expression of TRAIL R1 was observed in synovial tissues from patients with active RA, OA or SpA when compared with synovial tissues from either normal subjects or RA patients with inactive disease (p < 0.05; Table 2). TRAIL R1 was expressed mostly in the cytoplasm of cells in the synovial sublining and was virtually absent in synovial tissues from normal subjects (Figure 1). Expression of TRAIL R2, another TRAIL death receptor, was also significantly higher in the SpA group and active RA tissues compared with normal synovial tissues (p < 0.05; Table 2) with most staining associated with the nucleus (Figure 1). Although a similar trend was observed with the decoy receptor TRAIL R3 the differences were not as marked (Table 2; Figure 1). The other decoy receptor, TRAIL R4, was expressed in synovial tissues from all forms of arthritis and, to a lesser extent, in normal synovial tissues (Figure 1). TRAIL R4 was expressed predominantly in the synovial lining and appeared to be associated with the nuclear and perinuclear regions of cells, consistent with a previous report [41]. Statistical analysis for SQAs of all immunohistochemical labelling and TUNEL results is presented in Table 2.

**Table 2:** Semiquantitative analysis scoring for TRAIL, TRAIL receptors, TUNEL, cleaved caspase-3, survivin and xIAP

	**Normal**	**Active RA**	**Inactive RA**	**OA**	**SpA**
**TRAIL**	1.0 (0 to 3)	3.0 (1 to 4) * † ‡	2.0 (1 to 3)	2.0 (1 to 3)	2.0 (1 to 4) *
**TRAIL R1**	0 (0 to 2)	3.0 (0 to 4) * † ‡	1.0 (0 to 1)	2.0 (1 to 2) * †	2.0 (0 to 3) * †
**TRAIL R2**	0 (0 to 3)	2.0 (0 to 4) *	1.0 (0 to 3)	2.0 (0 to 3)	3.0 (1 to 3) *
**TRAIL R3**	2.0 (0 to 3)	4.0 (1 to 4)	2.0 (0 to 4)	3.0 (1 to 4)	4.0 (3 to 4)
**TRAIL R4**	1.0 (0 to 2)	3.0 (1 to 4) * ‡	3.0 (1 to 4) *	2.0 (1 to 3)	3.0 (2 to 4) *
**TUNEL**	0 (0 to 2)	0 (0) †	2.5 (0 to 3)	1.0 (0 to 2)	1.5 (0 to 3)
**Caspase-3**	0 (0 to 1)	3.0 (1 to 3) * † ‡	1.0 (0 to 1)	1.0 (0 to 1)	2.0 (0 to 2) * †
**Survivin**	2.0 (1 to 2)	3.0 (2 to 3) * † ‡	2.0 (1 to 2)	2.0 (1 to 2)	2.5 (0 to 3)
**xIAP**	0.0 (0 to 1)	2.5 (0 to 3)*	1.0 (0 to 1)	2.0 (0 to 3)	0.5 (0.0 to 2.5)

Results are presented as median scores (range).

* p < 0.05 compared with normal synovial tissue

† p < 0.05 compared with inactive rheumatoid arthritis (RA) synovial tissue.

‡ *p *< 0.05 compared with osteoarthritis (OA) synovial tissue.

SpA = spondyloarthropathies; TRAIL = tumour necrosis factor related apoptosis inducing ligand; TUNEL = terminal deoxynucleotidyl transferase biotin-dUTP nick end labelling; xIAP = x-linked inhibitor of apoptosis protein.

### Apoptosis detection

**Figure 2:**
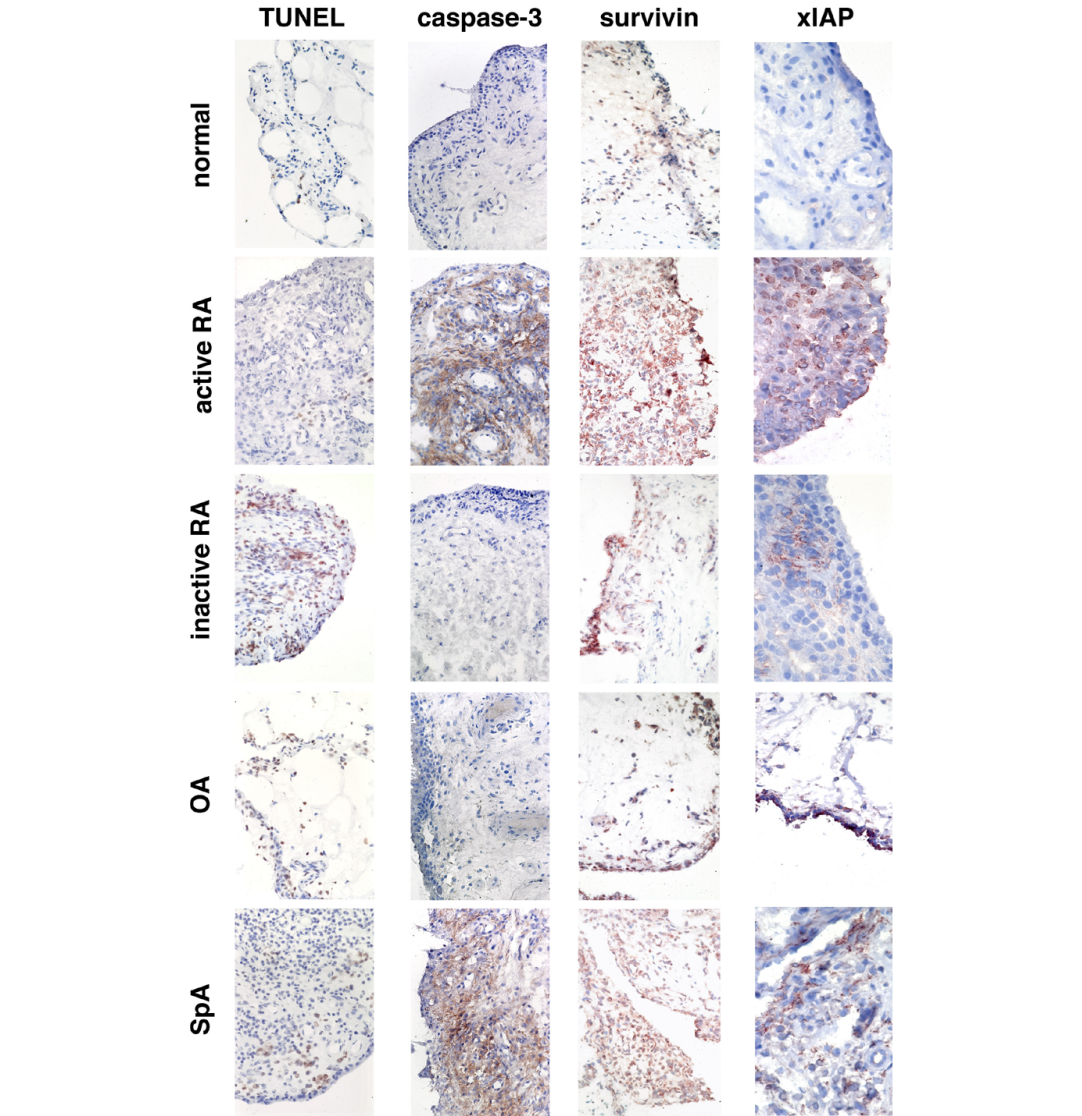
TUNEL, cleaved caspase-3, survivin and xIAP protein expression (red) in five patient groups. Magnification ×200, except for x-linked inhibitor of apoptosis protein (xIAP) × 400. OA = osteoarthritis; RA = rheumatoid arthritis; SpA = spondyloarthropathies; TUNEL = terminal deoxynucleotidyl transferase biotin-dUTP nick end labelling.

The TUNEL method identified many apoptotic cells in inactive RA synovium (Figure 2) but very few apoptotic cells were seen in active RA and normal synovia. This difference was statistically significant (p < 0.05; Table 2). Conversely, fewer cells expressing cleaved caspase-3 were observed in inactive RA but there were many cells expressing cleaved caspase-3 in active RA and SpA synovia (Figure 2). The difference between active and inactive RA was statistically significant (p < 0.05; Table 2). Cleaved caspase-8 was only weakly detected in the synovial tissues both from active RA and inactive RA patients (data not shown).

### Survivin and xIAP expression

**Figure 3:**
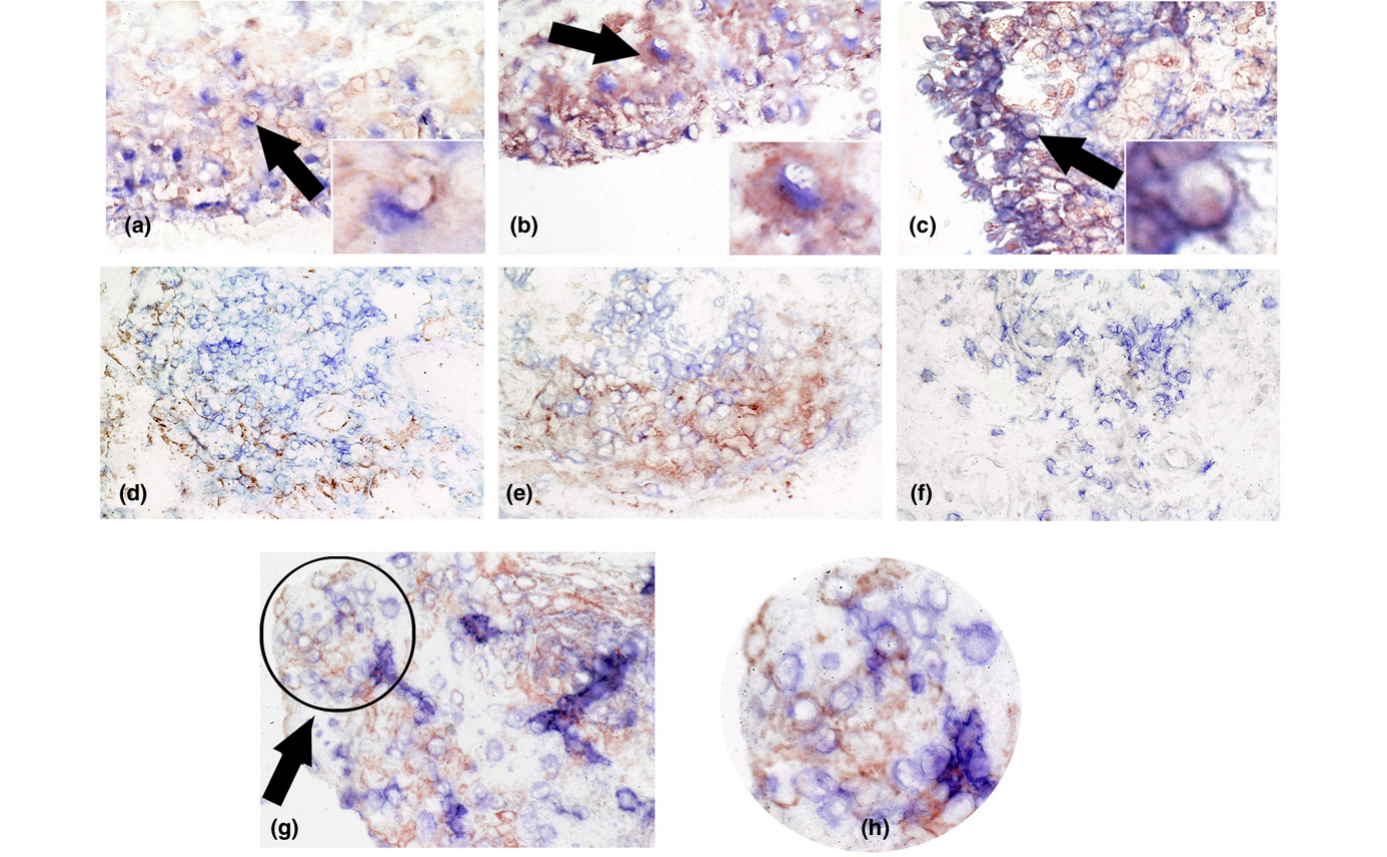
Double labelling of active RA tissues for expression of **(a)** TRAIL (red) and CD68 (blue); **(b)** TRAIL R1 (red) and CD68 (blue); **(c)** TRAIL R4 (red) and CD68 (blue); **(d)** TRAIL (red) and CD3 (blue); **(e)** TRAIL R1 (red) and CD3 (blue); **(f)** TRAIL R4 (red) and CD3 (blue). An arrow indicates from where the magnified image at the bottom right corner of panel was obtained in panels a-c. Panel g shows double labelling of cleaved caspase-3 and xIAP. Panel h is a magnified region of the image that is indicated by the arrow and circle in panel g. Magnification of panels is × 200 and magnified areas are approximately × 600.

Both xIAP and survivin were highly expressed in the cytoplasm of cells in synovial tissue from active RA (Figure 2). xIAP was expressed at significantly higher levels in active RA compared with normal synovial tissue (p < 0.05; Table 2). In addition, survivin was expressed at significantly higher levels in synovial tissue from patients with active RA compared with other types of synovial tissue tested (p < 0.05; Table 2; Figure 2). Dual labelling of this tissue for cleaved caspase-3 and xIAP demonstrated that many, but not all, cells expressing xIAP also expressed cleaved caspase-3 (Figure 3g,h).

### Cells expressing TRAIL and TRAIL receptors

Double labelling was carried out to determine the phenotype of cells expressing TRAIL and TRAIL receptors R1 and R4 (Figure 3). CD68 positive cells present in the synovial lining and sublining strongly expressed TRAIL (Figure 3a). Many CD68 negative cells expressed TRAIL R1 both in the synovial lining and sublining (Figure 3b). Like TRAIL, the decoy receptor TRAIL R4 was associated with CD68-positive cells mainly in the synovial lining (Figure 3c). CD3 positive lymphocytes expressed TRAIL R1 and, to a lesser extent, TRAIL (Figure 3e,d). TRAIL R4 was poorly expressed by CD3 positive cells (Figure 3f). Few cells expressing CD22 (B lymphocytes) or CD55 (synovial fibroblasts) also expressed TRAIL, TRAIL R1 or TRAIL R4 (data not shown). Double labelling of TRAIL R2 or TRAIL R3 with CD68 showed similar co-expression to that seen for TRAIL/TRAIL R1and CD68 (data not shown).

Strong correlations were observed between TRAIL and TRAIL R1 SQAs (r = 0.539, p < 0.0001). In addition, TRAIL R1 SQA was significantly correlated with CRP (r = 0.342, p < 0.009) and DAS28 (r = 362, p < 0.05). Also, a significant correlation was noted between survivin and cleaved caspase-3 SQAs (r = 0.492, p = 0.031) and between xIAP and survivin SQAs (r = 0.527, p = 0.005).

### Real-time PCR

**Figure 4:**
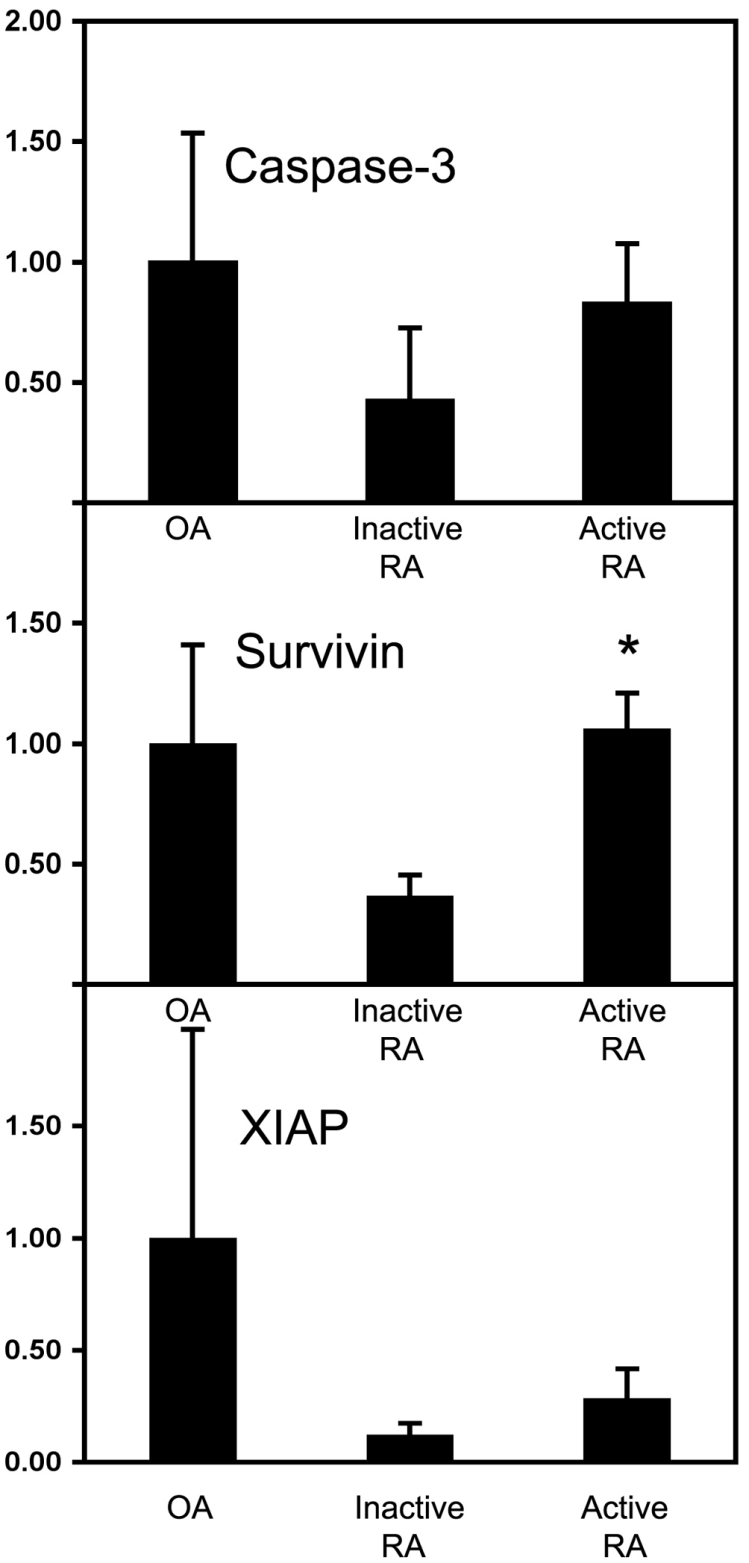
Expression levels of caspase-3, survivin and xIAP mRNAs in inactive RA, active RA and OA patients. These levels were determined by real-time PCR. The results are normalised to levels of hARP and have been expressed relative to OA control tissue. *p < 0.05, compared with inactive RA. n = 3 (OA), n = 3 (inactive RA), n = 5 (active RA). OA = osteoarthritis; PCR = polymerase chain reaction; RA = rheumatoid arthritis; xIAP = x-linked inhibitor of apoptosis protein.

Overall the relative levels of caspase-3, survivin and xIAP mRNA present in the different synovial tissues reflected the levels of protein determined immunohistologically above. The levels of caspase-3 mRNA expression in normal synovia and active RA revealed similar levels of expression, while there was a trend towards reduced caspase-3 expression with inactive RA (Figure 4), although this difference was not shown to be significant.

A similar pattern was found with survivin mRNA expression to that of caspase-3. There was a similar level of expression between normal synovia and active RA; however, survivin mRNA was expressed at significantly lower levels in inactive RA compared with active RA (p < 0.05, Figure 4).

xIAP mRNA expression was found to be highest in normal synovia compared with inactive and active RA; however, this expression was found to be quite variable within the samples investigated (Figure 4). In addition, a trend towards higher xIAP mRNA expression in active RA compared with inactive RA was also found (Figure 4), although a significant difference was not observed.

## Discussion

To the authors' knowledge, this is the first comprehensive study to demonstrate the expression of TRAIL and TRAIL receptors (both death and decoy receptors) in the synovial tissue from patients with a range of inflammatory and non-inflammatory arthritides, as well as in normal synovial tissue. Although OPG was not studied here, our previous work using tissue from a similar group of patients show that expression of this possible decoy TRAIL receptor was significantly reduced in active RA [32]. However, OPG was only expressed by vascular endothelial cells and some synovial macrophage populations, whereas the other TRAIL receptors studied here were widely expressed by infiltrating leucocytes.

This report demonstrates a marked increase in TRAIL expression in synovial tissue from patients with several types of arthritis that was largely due to the increased numbers of macrophages expressing TRAIL in the inflamed synovial tissues. Due to the co-expression of both death and decoy receptors in macrophages as seen in our finding, the net effect of this TRAIL upregulation on synovial tissue inflammatory cells is difficult to predict. Several studies have shown that TRAIL-induced apoptosis is regulated by the balance of the death and decoy receptors expressed by cells [42-44] and the relative expression of TRAIL death or decoy receptors may be important in regulating apoptosis in active arthritides. However, while other upstream molecules stimulating apoptosis may be involved, the presence of cleaved caspase-3 in active RA synovial tissues observed in our study is consistent with the possibility of TRAIL binding to death receptor(s) resulting in downstream signalling of apoptosis.

The elevation of TRAIL expression is consistent with a recent report showing that soluble TRAIL levels are higher in RA synovial fluid compared with OA synovial fluid [45]. In contrast to the results reported here, Perlman and colleagues [27] were unable to detect elevation of TRAIL or TRAIL receptors in synovial fibroblasts but did observe increased TRAIL R3 in RA synovial fluid macrophages. The different findings may have resulted from differences in the source of the samples (cultured cells versus intact synovium), technique (flow cytometry versus histology) and also the different antibodies used in each study. A study by Ichikawa and colleagues [18] was performed both on synovial fibroblasts in culture and on the synovial membrane but was limited to the investigation of only TRAIL R1 and TRAIL R2. Their study reported that only TRAIL R2 was expressed in the synovial tissues *in situ *but not TRAIL R1.

We noted high expression of TRAIL R1 and R2 in the cytoplasm and perinuclear regions of the cell, respectively. In contrast the cell surface membrane only weakly stained for the presence of these receptors. This is consistent with studies of apoptosis in melanoma cells that have reported nuclear localisation of TRAIL R2 and may be one possible mechanism of escape from apoptosis [46].

The discrepancy between TRAIL and TRAIL receptor expression on cultured synovial fibroblasts and that seen in the synovial membrane highlights the importance of studying the expression of TRAIL and its receptors comprehensively in the intact synovial membrane, as undertaken in this present study. Studies with synovial fibroblasts are limited because of the presence in the inflamed synovial tissues of many lymphocytes and cells of the macrophage/monocyte lineage. The overwhelming number of non-fibroblastic inflammatory cells are important in regulating the progression of RA. Therefore, regulation of apoptosis in these cells may be a key process that modulates disease. Remission in RA patients, particularly after DMARD treatment, has been reported to be associated with a decrease in macrophage content of the synovium [47].

It was expected that the marked elevation in the levels of TRAIL and TRAIL death receptors observed in the active RA synovial tissues would result in an increase in apoptosis. However, this was not the case, with very few TUNEL positive cells being observed in active RA synovial tissue despite high levels of activated caspase-3. Although events upstream of activation of caspase-3 were not investigated and may play a role, the expression of high levels of caspase-3 in active RA indicate that inhibition of apoptosis may largely occur downstream of caspase-3 activation. Furthermore, inhibitors of activated caspase-3, such as the IAP family members, survivin and xIAP, are likely to be involved because there was a significant correlation between survivin and cleaved caspase-3 expression. Additionally, our finding that the mRNAs of caspase-3, xIAP and survivin were generally higher in active RA compared with inactive RA synovial tissue is consistent with the protein data. This further supports the contention that these inhibitors have a role in maintaining active RA. The increase of both xIAP and survivin in active RA synovial tissue may be even more significant because of the known synergy of survivin and xIAP forming a complex that promotes increased xIAP stability against ubiquitination/proteasomal destruction, as previously reported [48]. Other molecules may also be indirectly involved in the regulation of caspase-3. For example, SMAC/Diablo regulates the IAP family members and may indirectly regulate caspase-3 through this mechanism [49].

Unlike caspase-3, we observed low levels of caspase-8 in active RA synovial tissues. This could be either because of activation of caspase-3 by the intrinsic pathway through caspase-9 (instead of through TRAIL receptor activation and caspase-8) or activation of caspase-8 may be inhibited by the over expression of FLIP, which has been reported in active RA synovial tissue [50].

Although dual labelling was not technically possible due to the types of antibodies used, we did carry out staining of sequential sections of tissues and found that the same populations of cells expressing both TRAIL death receptors and xIAP/survivin (data not shown). In addition, our data presented here show that TRAIL death receptors and xIAP/suvivin are expressed by large numbers of CD68 positive cells in the synovium.

Although RA was the major focus of this study, OA and SpA synovial tissues were also investigated. As might be expected, tissue from the other inflammatory arthritis group, SpA patients had significantly elevated expression of TRAIL and TRAIL receptors compared with normal tissues, similar to the active RA tissues. This indicates that TRAIL R1 may be important in other inflammatory arthritides and is not specific to RA. The significant correlation between TRAIL R1, CRP and DAS28 observed in this study supports this hypothesis. However, it was surprising to observe a significant increase in TRAIL R1 expression in OA synovial tissues as OA is not usually associated with marked synovial inflammation. Although it is possible that this could be because of the effects of low-grade inflammation of the synovium that is associated with late stages of OA [49], it would be of interest to determine whether TRAIL is related to the OA disease process.

The regulation of apoptosis is complex with ligands, receptors and intracellular signalling pathways playing important roles in determining whether apoptosis occurs. Apoptosis in active RA may be important as a way of limiting inflammation by reducing the number of inflammatory cells and could potentially be a therapeutic target.

## Conclusion

This study indicates that the induction of apoptosis in active RA synovial tissues is inhibited despite stimulation of the intracellular pathway(s) that lead to apoptosis, possibly through TRAIL and TRAIL death receptors. In addition to the inhibition mediated by TRAIL decoy receptors, inhibition of apoptosis appeared to be downstream of caspase-3 and may involve the caspase-3 inhibitors survivin and xIAP. Although further *in vitro *studies involving culture of the appropriate inflammatory RA synovial cell types would be needed to confirm our findings, this study suggests that there are potential therapeutic targets downstream of caspase-3 cleavage that could be used to control active RA.

## Abbreviations

ACR: American College of Rheumatology; CRP: C-reactive protein; DAS28: disease activity score 28; DMARD: disease modifying antirheumatic drug; ESR: erythrocyte sedimentation rate; FADD: Fas associated death domain; FLIP: flice inhibitory protein; IAP: inhibitor of apoptosis protein; Ig: immunoglobulin; OA: osteoarthritis; OPG: osteoprotegerin; PCR: polymerase chain reaction; RA: rheumatoid arthritis; RF: rheumatoid factor; SpA: spondyloarthropathies; SQA: semiquantitative analysis; TNF: tumour necrosis factor; TRAIL: tumour necrosis factor related apoptosis inducing ligand; TUNEL: terminal deoxynucleotidyl transferase biotin-dUTP nick end labelling; xIAP: x-linked inhibitor of apoptosis protein.

## Competing interests

The authors declare that they have no competing interests.

## Authors' contributions

AASSKD and DRH were responsible for design of the study, interpretation of data and preparation of the manuscript. AASSKD carried out the immunohistology. MDS, DMF and AE contributed to the manuscript's intellectual content. MJA was responsible for statistical analysis and interpretation of data. PC, GS and XNX were responsible for development of some antibodies used in the study. CAH was responsible for acquisition, analysis and interpretation of RT PCR data. HW and MDS were responsible for acquisition of tissue and patient data.
